# Efficacy of Essential Oil Vapours in Reducing Postharvest Rots and Effect on the Fruit Mycobiome of Nectarines

**DOI:** 10.3390/jof10050341

**Published:** 2024-05-08

**Authors:** Giulia Remolif, Fabio Buonsenso, Giada Schiavon, Marco Garello, Davide Spadaro

**Affiliations:** 1 Department of Agricultural, Forest and Food Sciences (DISAFA), University of Turin, Largo Paolo Braccini 2, 10095 Grugliasco, Italy; gi.remolif@unito.it (G.R.); fabio.buonsenso@unito.it (F.B.); schiavon.giada@gmail.com (G.S.); marco.garello@unito.it (M.G.); 2 Interdepartmental Centre for the Innovation in the Agro-Environmental Sector—AGROINNOVA, University of Turin, Largo Paolo Braccini 2, 10095 Grugliasco, Italy

**Keywords:** biofumigation, *Prunus persica*, stone fruit, metabarcoding, brown rot

## Abstract

Nectarines can be affected by many diseases, resulting in significant production losses. Natural products, such as essential oils (EOs), are promising alternatives to pesticides to control storage rots. This work aimed to test the efficacy of biofumigation with EOs in the control of nectarine postharvest diseases while also evaluating the effect on the quality parameters (firmness, total soluble solids, and titratable acidity) and on the fruit fungal microbiome. Basil, fennel, lemon, oregano, and thyme EOs were first tested *in vitro* at 0.1, 0.5, and 1.0% concentrations to evaluate their inhibition activity against *Monilinia fructicola*. Subsequently, an *in vivo* screening trial was performed by treating nectarines inoculated with *M. fructicola*, with the five EOs at 2.0% concentration by biofumigation, performed using slow-release diffusers placed inside the storage cabinets. Fennel, lemon, and basil EOs were the most effective after storage and were selected to be tested in efficacy trials using naturally infected nectarines. After 28 days of storage, all treatments showed a significant rot reduction compared to the untreated control. Additionally, no evident phytotoxic effects were observed on the treated fruits. EO vapors did not affect the overall quality of the fruits but showed a positive effect in reducing firmness loss. Metabarcoding analysis showed a significant impact of tissue, treatment, and sampling time on the fruit microbiome composition. Treatments were able to reduce the abundance of *Monilinia* spp., but basil EO favored a significant increase in *Penicillium* spp. Moreover, the abundance of other fungal genera was found to be modified.

## 1. Introduction

Nectarines (*Prunus persica* var. *nucipersica*) are highly appreciated fruits for their taste and high nutritional value. However, they are perishable products and highly susceptible to fungal infections. The most important pathogens are *Monilinia fructicola*, *M. laxa*, and *M. fructigena*, the causal agents of brown rot, which lead to significant yield losses and reduced shelf life [1,2]. Additionally, other fungal pathogens such as *Botrytis cinerea*, *Rhizopus* spp., and *Penicillium expansum* can cause minor losses on stone fruits [2]. Although control of major postharvest pathogens can be achieved with the preharvest application of synthetic fungicides, there are growing concerns about the human health and environmental risks associated with pesticide use, along with the development of fungicide-resistant strains. Therefore, it is necessary to develop new and effective control methods that are safe and less risky to consumer health and the environment [1,3]. These include the use of physical means, such as hot-water dipping [4], biological control agents [5], and natural compounds, such as extracts from plants and microbes and secondary metabolites [6]. In this context, essential oils (EOs) could represent a management strategy with important and not entirely known potential [7,8,9]. Essential oils are highly concentrated, volatile, aromatic liquids derived from plants. They are popular for their wide range of applications, including aromatherapy, personal care products, natural cleaning agents, and even culinary purposes [10,11,12]. Moreover, their antimicrobial and antioxidant properties [13,14] make them attractive for preserving the quality and extending the shelf life of fruits and vegetables [3,15,16]. EOs are extracted from various plant parts, including flowers, leaves, bark, stems, and roots [17]. Extraction methods commonly used include steam distillation, cold-press extraction, and solvent extraction [18,19]. Non-conventional methods, such as the use of supercritical fluids like CO_2_ [20,21] or solvent-free microwave extraction [22,23], can also be employed to obtain EOs. The chemical composition of EOs is complex and can vary depending on the plant species (botanical source and plant part used) and other factors, like growing conditions, harvesting time, extraction method, storage, and aging [24,25]. In fact, exposure to heat, light, and air can cause oxidation and degradation of some compounds over time, altering the overall profile [26,27]. Each EO has its own unique composition of chemical constituents, giving it distinct properties and potential benefits. EOs are composed of a wide array of volatile organic compounds (VOCs), including terpenes, in particular, mono- and sesquiterpenes, esters, alcohols, ketones, phenols, and others [17,28].

*In vitro* and *in vivo* studies demonstrated the antifungal activity of EOs against fruit postharvest pathogens, such as *Botrytis cinerea* [29,30,31], *Penicillium expansum* [32,33], *Alternaria* spp. [34], and *Monilinia* spp. [31,35]. The antimicrobial activity of EOs has been associated with several mechanisms, including the alteration in cell wall and membrane permeability and changes in gene expression patterns [36]. Additionally, EOs have been shown to induce host resistance through the priming host defense responses [37]. The use of these substances in managing postharvest diseases, however, is hampered by phytotoxicity phenomena even at moderate concentrations [30]. A possible solution is the application of EOs through biofumigation, in which they are released slowly into the storage atmosphere through diffusers, avoiding direct contact with the fruits [16,33,38].

The aim of this study was to investigate the effect of the vapour treatment of basil, fennel, lemon, oregano, and thyme EOs, applied using biofumigation through slow-release diffusers, in the control of nectarine postharvest diseases. *In vitro* and *in vivo* screening trials were performed to identify the most effective EOs against *Monilinia fructicola*. The selected EOs were subsequently tested in efficacy trials using naturally contaminated nectarines while assessing their impact on the fruit quality parameters. Finally, the effect of the postharvest application of basil EO on the epiphytic and endophytic fungal microbiome of nectarines was assessed.

## 2. Materials and Methods

### 2.1. Essential Oils

Organic basil (*Ocimum basilicum*), fennel (*Foeniculum vulgare* var. *dulce*), lemon (*Citrus limon*), oregano (*Origanum vulgare*), and thyme (*Thymus vulgaris*) EOs used in this work were purchased by Flora Srl (Lorenzana, Pisa, Italy; certifications quality assurance: UNI EN ISO 9001 and 14001), part of a range of products referred to as “*Primavera Essential oils BIO*”. The chemical compositions of the five EOs were analyzed through gas chromatography coupled with mass spectrometry (GC-MS) in our previous paper [33].

### 2.2. In Vitro Biofumigation Assays

The antifungal activity of the selected EOs was preliminarily evaluated *in vitro* against *Monilinia fructicola* using the sandwich-plate method. The essential oils were tested at 0.1%, 0.5%, and 1.0% (*v*/*v*) concentrations. Potato Dextrose Agar (PDA, VWR International, Leuven, Belgium) medium was poured into 90 mm diameter Petri dishes (15 mL per Petri dish), and the percentage of EOs tested was added after autoclaving. Petri dishes with only PDA medium were inoculated with a mycelium plug (8 mm diameter) obtained from 15-day-old cultures of *M. fructicola* strain CVG1539, taken from the collection of the University of Turin. Petri dishes with the EOs were placed upside down over the Petri dishes inoculated with *M. fructicola* to build a sandwich. The plates were sealed immediately with Parafilm and incubated at 25 ± 1 °C for 28 days. The diameter of fungal growth was measured every 14 days. Controls were set up using PDA Petri dishes without EOs on the top of inoculated Petri dishes. The assay was set up with five biological replicates and performed twice.

### 2.3. Efficacy of EOs In Vivo

#### 2.3.1. Screening Trial

A screening trial was set up on nectarines cv. Big Top using thyme, basil, oregano, fennel, and lemon EOs at 2.0% (*v*/*v*) concentration. The concentration used was selected after previous trials conducted on nectarines. Three replicates of 10 nectarines were prepared for each treatment. Fruits, selected without evident wounds and rots, were disinfected in a 1.0% sodium hypochlorite solution, rinsed in tap water, dried, and wounded using a sterile tip (3 mm depth) at the equatorial region.

For inoculum preparation, *M. fructicola* strain CVG1539 was cultured on PDA amended with 25 mg/L of streptomycin (Merck, Darmstadt, Germany) for 15 days at 25 ± 1 °C under 12 h photoperiod. Once conidial fructification was obtained, 10 mL of a 1.0% Tween-20 solution was added to each plate, and conidia were collected using an L-shaped spatula. The quantification was carried out using a Burker chamber, and the suspension was brought to a final concentration of 1 × 10^4^ conidia/mL. Inoculation was performed by dipping the fruits in the conidial suspension. Biofumigation was performed using slow-release diffusers made of EO gel emulsions that release EOs vapor phase during storage. Diffusers were prepared by adding EO (2.0%) in sterile deionized water (97%) and Tween-20 (1.0%). The solutions were then poured into 90 mm Petri dishes (15 mL per Petri dish). Plastic boxes containing the nectarines were placed in sealed storage cabinets with six slow-release diffusers. The diffusers were placed open inside the cabinets and positioned around the boxes. Three controls were included: a chemical control, inoculated with *M. fructicola* and treated with cyprodinil and fludioxonil (Switch^®^, a.i. 37.5% and 25.0%, Syngenta Italia S.p.A., Milan, Italy), an inoculated control (inoculated and not treated) and a healthy control (not inoculated and not treated). Disease incidence was evaluated after 14 days of storage in a normal atmosphere at 1 ± 1 °C and 95% RH, then again after 7 days of shelf life at 20 ± 1 °C without the presence of the diffusers.

#### 2.3.2. Efficacy Trial

Basil, lemon, and fennel EOs were selected for setting up an efficacy trial, in which they were used at 2.0% (*v*/*v*) concentration. For each treatment, three replicates of 30 nectarines were set up. The pathogen was not inoculated to evaluate the effectiveness of the treatments on naturally occurring infections. The preparation of EO diffusers was carried out according to the procedures described for the screening test. A chemical control treated with cyprodinil and fludioxonil, as previously reported, and an untreated control were included. Evaluation of disease incidence was performed after 28 days of storage at 1 ± 1 °C in a normal atmosphere and 95% RH with EO diffusers and then after 5 days of shelf life at 20 ± 1 °C in a chamber without EO diffusers.

#### 2.3.3. Quality Analyses

Fruit quality analyses were carried out by measuring firmness, total soluble solids, and titratable acidity. Values were determined at harvest, at the end of the storage, and at the end of shelf life. Analyses were performed using three replicates of five nectarines for each treatment.

Firmness (N/cm^2^) was measured using the Fruit Texture Analyzer (FTA, Turoni, Italy) with an 8 mm tip on two opposite points of the fruit’s equatorial region after removing the skin.

The total soluble solids content was determined using the refractometer NR151 (Rose Scientific Ltd., Edmonton, AB, Canada). For the measurements, juice was obtained from the nectarines using a juice extractor. Values obtained were expressed as the percentage of soluble solid content.

The titratable acidity was determined using the extracted juice. For each replicate, 6 g of juice was weighed, added with 50 mL of water, and subsequently titrated with 0.1 N NaOH up to a final pH value of 8.2, measured by using a FiveEasy Plus pH meter FP20-Std-Kit (Mettler Toledo, Milan, Italy).

The titratable acidity was calculated using the following formula:

(V_NaOH_ × 0.0067 × 100)/6

where 0.0067 indicates the acidity factor of the malic acid, and the value 6 represents the grams of juice used for the titrating solution.

### 2.4. Statistical Analyses

Statistical analyses were performed using IBM SPSS software version 27.0 (SPSS Inc., Chicago, IL, USA). Data were subjected to analysis of variance (ANOVA), and statistical significance was assessed at the level of *p* < 0.05. Duncan’s multiple range test was used to separate the means.

### 2.5. Microbiome Sampling, Sequencing and Bioinformatic Analyses

Sampling of the fruit microbiome was performed at harvest, after 28 days of storage at 1 ± 1 °C and after 5 days at 20 ± 1 °C (shelf life). Based on the efficacy results of the previous experiments, only fruit treated with basil essential oil, in addition to the untreated control and chemical control, were selected for sampling. Five biological replicates consisting of 15 nectarines were analyzed at each time point for each treatment. Microbiome sampling and genomic DNA extraction were performed as described in Schiavon et al. [16]. For epiphyte sampling, the surface of each nectarine was rubbed with sterile cotton swabs dipped in sterile phosphate-buffered saline (PBS) solution. Cotton swab tips belonging to the same replicate were collected in a sterile tube with 8 mL of PBS solution. Tubes were shaken at 250 rpm for 20 min and sonicated for 5 min at 40 kHz. Swab tips, squeezed with sterile tweezers, were then transferred into new tubes containing 4 mL of PBS solution, shaken manually, and squeezed. Suspensions of the first and second tubes were pooled and centrifuged at maximum speed for 30 min. The resulting pellet was resuspended in 2 mL of PBS buffer and centrifuged again with the same conditions. The obtained pellet was stored at −20 °C until DNA extraction. 

For endophyte sampling, nectarines were washed in 5% bleach for 2 min, rinsed twice in water for 2 min, and allowed to dry on sterile paper. The fruit surface was then rubbed with ethanol-soaked cotton to remove residual epiphytic contaminations. Sections of peel, each 1 cm wide, were removed all around the equator of each fruit and placed in sterile plastic jars. Samples were lyophilized, frozen in liquid nitrogen to obtain a dry powder, and stored at 20 °C until DNA extraction. 

DNA extraction of epiphytes was performed using the Wizard Genomic DNA Purification Kit (Promega Biotech AB, Finnboda Varvsväg, Sweden), following the manufacturer’s instructions, while DNA extraction of endophytes was performed using the DNeasy Power Soil Pro kit (Qiagen, Hilden, Germany), following the manufacturer’s protocol with minor modifications [16].

Library preparation, pooling, and sequencing were performed at the IGA Technology Services facility (IGATech, Udine, Italy). Libraries were prepared by following Illumina 16S Metagenomic Sequencing Library Preparation protocol [39] in two amplification steps: an initial PCR amplification using locus-specific PCR primers and a subsequent amplification that integrates relevant flow-cell-binding domains and unique indices (NexteraXT Index Kit, FC-131-1001/FC-131-1002). For the locus-specific amplification, 29 cycles were applied in the first PCR reaction using primers fITS7b (GTGARTCATCGAATCTTTG [40]) and ITS4 (TCCTCCGCTTATTGATATGC [41]). Libraries were sequenced on a NovaSeq6000 instrument (Illumina, San Diego, CA, USA) using 250 bp paired-end mode. 

Sequence analysis was performed using the QIIME2 suite [42] and custom Python scripts. Adapter contamination was removed with the Cutadapt plugin [43]. Based on an assessment of the quality-over-length sequence profile, no sequence trimming was performed. Read merging, ASV generation, and chimera filtering were performed using DADA2 [44]. A naive Bayes predictor was trained on the UNITE 8.3 global database [45], integrated with other ITS sequences from the NCBI nucleotide database [46], and used to classify the previously generated ASVs. Reads not belonging to fungi, reads that present a global abundance of less than 0.05% of total sequences, and reads that appeared in less than four replications were removed. The Shannon diversity index and the number of observed features were selected as alpha diversity metrics, while the robust Aitchison distance was chosen as the beta diversity metric. The normalization of samples for alpha analysis was performed using the scaling with ranked subsampling (SRS) approach implemented in the module by the same name [47], with a normalization value of 12000. Both alpha diversity metrics were calculated with the default plugin, while beta diversity, normalization, calculation, and subsequent dimensional reduction were performed with the DEICODE plugin [48]. Statistical analyses of alpha diversity results were performed using the non-parametric Kruskal–Wallis test followed by a Dunn post hoc test with Benjamini–Hochberg correction. For significance, a q-value (FDR-adjusted *p*-value) threshold of 0.05 was selected <0.05. For beta analysis, a permutational analysis of variance (PERMANOVA) both with the Adonis plugin [49,50] and the default QIIME2 plugin (for pairwise comparisons, as well as a permutational dispersion analysis (PERMDISP) with the default QIIME2 plugin were performed. Once more, a q-value (FDR-adjusted *p*-value) threshold of 0.05 was selected. Compositional analyses were performed using custom Python scripts. ASV absolute frequencies were collapsed at the genus level for each sample and converted to relative frequencies, and then samples were grouped based on time point, tissue, and treatment. To improve data readability, genera with less than 1.0% frequency across all groups were collapsed into the “other” category. Finally, data were plotted as histograms.

## 3. Results

### 3.1. In Vitro Biofumigation Assay

Mycelial growth of *M. fructicola* was measured after 14 and 28 days of exposure to EO vapors (Table 1).

After 14 days, thyme and oregano EOs at all concentrations used (1.0, 0.5, and 0.1%), fennel EO at 1.0% and 0.5%, and basil EO at 1.0% showed 100% inhibition of *M. fructicola* growth. Basil EO at 0.5% showed a non-complete inhibition but was not statistically different from the 1.0% treatment. Basil and fennel EOs at 0.1% and lemon EO at all tested concentrations could not significantly reduce the mycelial growth. Furthermore, the mycelial growth of 0.1% lemon EO was statistically comparable to the control. After 28 days, the data confirmed a complete inhibition of the mycelial growth for thyme EO at all tested concentrations, for oregano EO at 1.0% and 0.5%, and for basil and fennel EOs at 1.0%. Basil and fennel EOs at 0.1% and lemon at all tested concentrations were not effective in reducing the mycelial growth of *M. fructicola*, as no significant differences were observed compared with the control.

### 3.2. Efficacy of EOs In Vivo 

#### 3.2.1. Screening Trial

**Figure 1:**
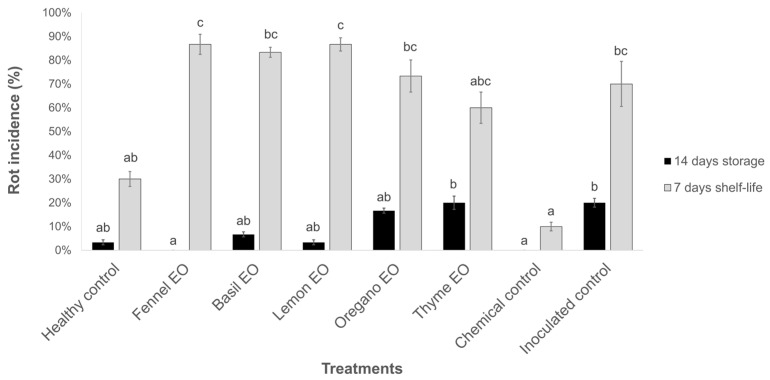
Rot incidence (%) ± standard error (SE) on nectarines treated with essential oil biofumigation stored at 1 ± 1 °C for 14 days and kept in shelf life at 20 ± 1 °C for 7 days. Values at the same time point, followed by the same letter, are not statistically different by Duncan’s multiple range test (*p* < 0.05).

The *in vivo* screening trial was performed by treating nectarines inoculated with *M. fructicola* with the five EOs at 2.0% concentration (Figure 1). 

After 14 days of storage at 1 ± 1 °C, no observable rots developed on fruits treated with fennel EO, as well as in the chemical control. Among the other treatments, basil, oregano, and lemon EOs showed less than 10% disease incidence, although not statistically different from the inoculated control. After shelf life, without EO diffusers, all previously treated fruits showed a rot incidence not statistically different from the inoculated control, with values greater than (basil, fennel, lemon, and oregano) or equal to (thyme) 60%. Considering the results obtained after storage, fennel, lemon, and basil EOs were selected to be tested in the efficacy trial using naturally contaminated nectarines.

#### 3.2.2. Efficacy Trial

**Figure 2:**
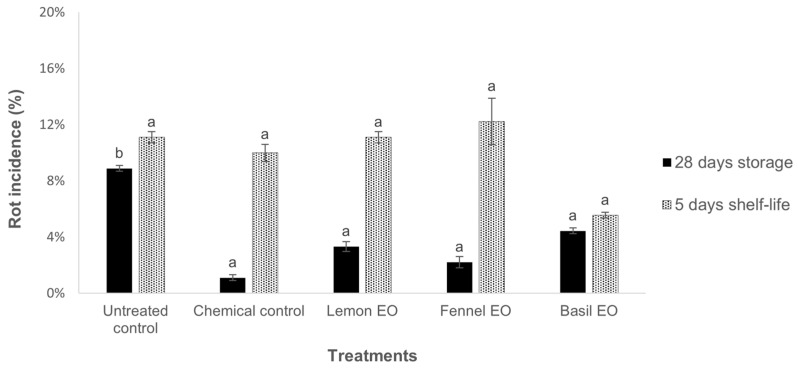
Rot incidence (%) ± standard error (SE) on nectarines treated with essential oil biofumigation stored at 1 ± 1 °C for 28 days and kept in shelf life at 20 ± 1 °C for 5 days. Values at the same time point, followed by the same letter, are not statistically different by Duncan’s multiple range test (*p* < 0.05).

The trial on naturally infected fruits showed the effectiveness of the three tested EOs—fennel, basil, and lemon—in reducing the rot incidence after 28 days of storage at 1 ± 1 °C (Figure 2). The treatments showed a rot incidence significantly lower compared to the untreated control, comparable to the chemical treatment. After 5 days of storage, in which the fruits were no longer exposed to EO vapors, no statistical differences were observed when comparing the rot incidence of the treated fruits with the untreated control, showing that the treatments were no longer able to control the disease development.

#### 3.2.3. Quality Analyses

Quality analyses performed at harvest, after storage, and after shelf life showed a decrease in firmness and titratable acidity and an increase in total soluble solids over time (Table 2).

Significant differences were observed in firmness at the end of shelf life, as fruits treated with EOs had slightly higher firmness than the untreated control. No significant differences were observed in titratable acidity and total soluble solids content for treated and control fruits at all time points.

### 3.3. Microbial Diversity and Composition

**Figure 3:**
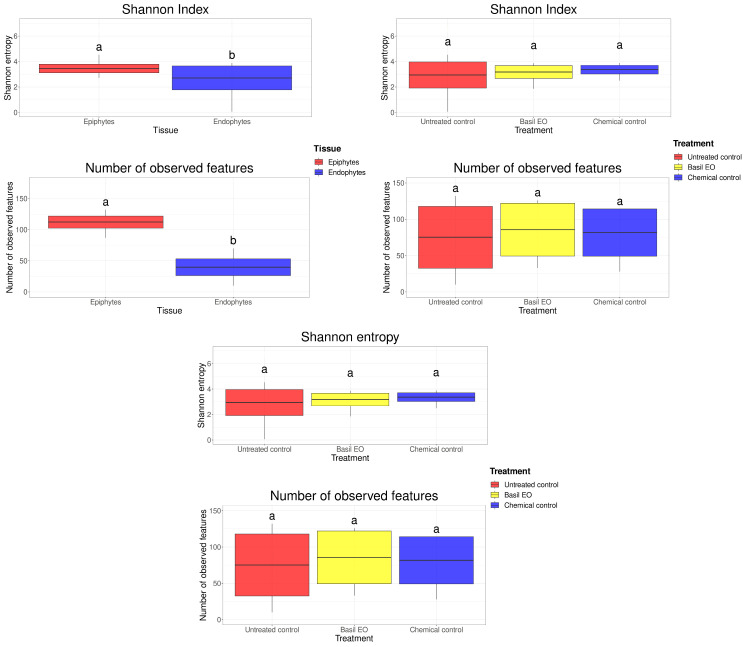
Box and whisker plots of alpha diversity values of analyzed microbial communities based on tissue, sampling time point, and treatment group. The middle line of each box coincides with the mean, while the upper and lower box bounds are placed at one standard deviation from the mean. Whiskers above and below the box extend to the highest and lowest values of their respective group. Letters above each plot indicate the significance group of that plot. Comparison of sample groups and assignation to significance groups was performed by means of a Kruskal–Wallis test followed by a Dunn post hoc test, with a q-value (FDR-adjusted *p*-value) rejection threshold set at 0.05.

By considering the alpha diversity (Figure 3), the comparison between the sampled tissues, skin, and pulp showed the presence of a significant difference for both the Shannon index and the number of observed features, with epiphytes having higher values for both metrics than endophytes. The comparison between the time points did not show any significant difference between groups for the number of observed features, while a significant difference was found between harvest and the other time points for the Shannon index. The treatments considered did not show any significant difference between groups either for the Shannon index or the number of observed features.

**Figure 4:**
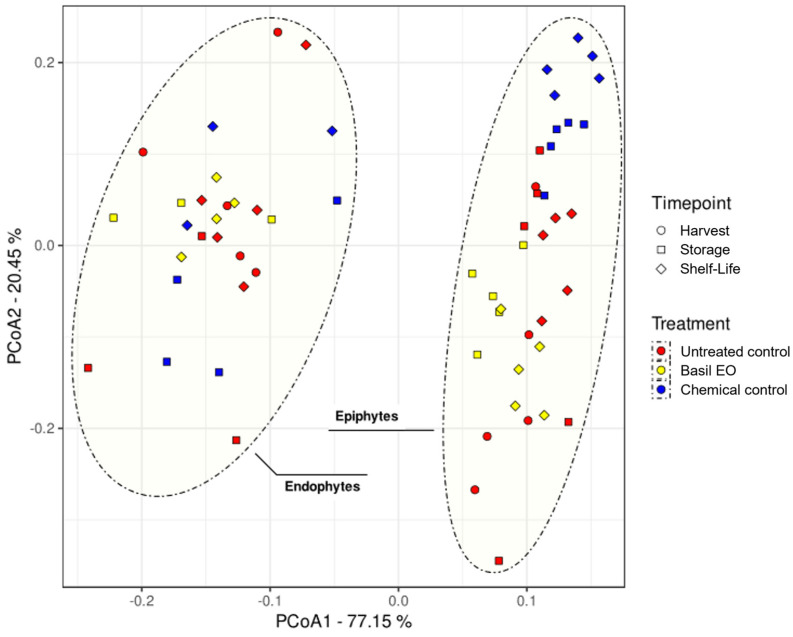
PCoA plot of the robust Aitchison distance matrix values calculated on analyzed samples. Percentage values represented on the main axes indicate explained variance associated with each principal component. Point shape indicates the sampling time point, while color indicates the associated treatments. Finally, group ellipses indicate the associated tissue.

The beta diversity was described by the Adonis analysis (Table S1) and by the sample distribution in a low-dimensional space following the PCoA analysis (Figure 4). Adonis analysis showed a statistically significant impact of tissue, treatment, and sampling time point, as well as all pairwise combinations of parameters on the total variance. Among the parameters, tissue had the highest impact (44% of total variance), followed by treatment (11%) and sampling time point (4%), while pairwise parameter combinations ranged from 9% (tissue × treatment) to 4% (tissue × sampling time point and treatment × sampling time point).

Samples presented a stark separation based on tissue variance (Figure 4). In the same plot, the effect of treatment is also visible, but only for the epiphytic communities. These observations are confirmed by the pairwise PERMANOVA and PERMDISP (Tables S2 and S3). The results of PERMANOVA and PERMIDISP also showed that the sampling time point had the lowest impact among the considered factors, as it only impacted the epiphytic communities and only for fruit treated with basil EO or the fungicide.

**Figure 5:**
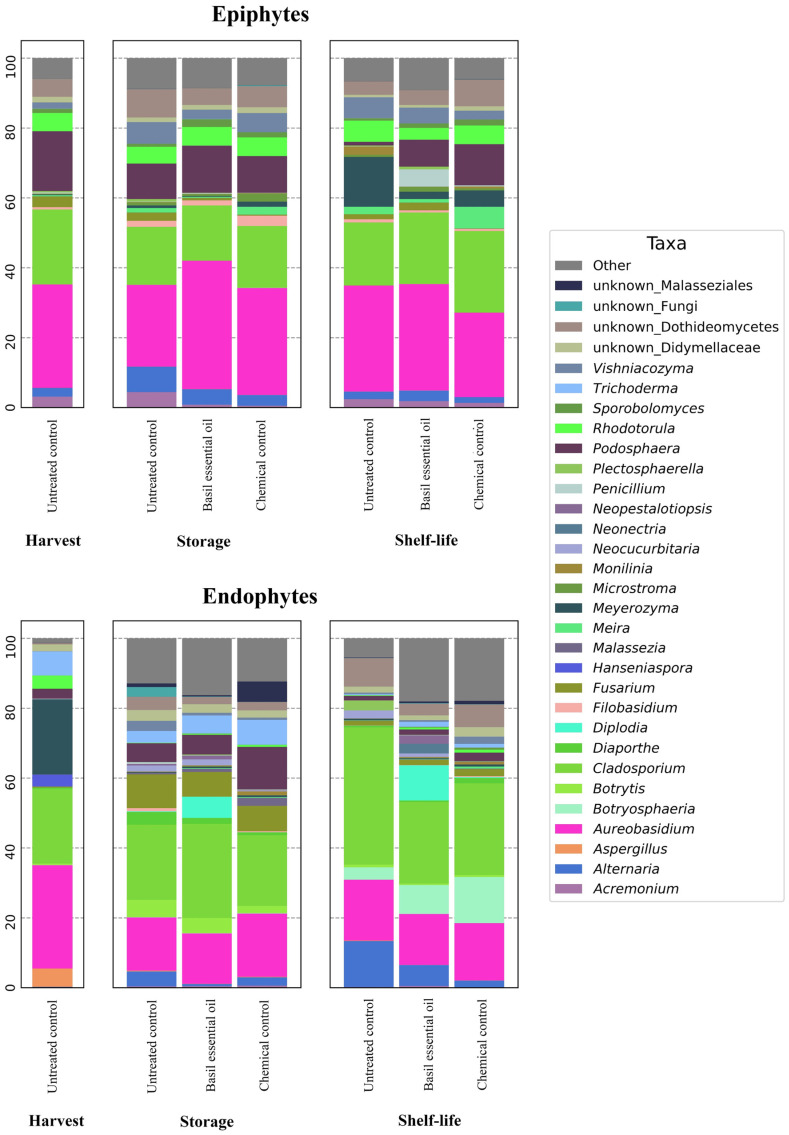
Taxa compositions of epiphytic (upper side) and endophytic (lower side) fungal communities. The “Other” category includes all taxa with less than 1% relative presence in all considered groups.

Focusing on the composition of the epiphytic population (Figure 5), *Monilinia* spp. had a lower abundance during shelf life in chemical control (0.1%) and EO treatment (0.5%) compared to untreated fruit (2.4%). In fruit treated with basil EO, a higher presence of *Penicillium* spp. was registered (5.0%) compared to the other treatments.

The occurrence of *Podosphaera* was reported at harvest (17.1%) and at the end of the storage, with similar values across treatments (10.1% for the untreated control, 10.5% for the chemical control, and 13.5% for the EO treatment). The presence of this genus decreased at the end of shelf life, with the highest abundance in the fungicide treatment (12.0%), followed by the EO treatment (7.8%), and the untreated control (1.0%). Among the non-pathogenic fungi, a high abundance of *Aureobasidium* was found in all treatments at all time points, particularly on fruit treated with basil EO at the end of storage (36.7%). Other yeast genera presented a similar stable abundance across time point and treatment, in particular, *Vishniacozyma* (1.7–6.2%) and *Rhodotorula* (3.3–6.1%). In contrast, during shelf life, *Meyerozyma* presented a higher abundance in untreated fruit (14.2%) compared to both chemical control (4.7%) and EO treatment (2.1%).

In the endophytic population, a high occurrence of *Diplodia* was found in fruit treated with basil EO, both after storage (6.0%) and at the end of shelf life (10.0%). *Podosphaera* was present in all treatments at the end of the storage, with higher values for the chemical treatment (12.2%) compared to untreated control and EO treatment (5.4% and 5.6%, respectively). *Botryosphaeria* was found in all treatments in shelf life, with higher values for chemical (13.2%) and basil EO (8.3%) treatments compared to untreated control (3.5%). A slight increase in *Diaporthe* was observed in the untreated fruits at the end of the storage (3.7%). *Botrytis* was detected at the end of the storage in untreated control (4.9%) and less abundant in chemical- (2.1%) and EO-treated (4.3%) nectarines. *Alternaria* was found in stored fruit (0.6% for the EO treatment, 2.4% for the chemical control and 4.3% for the untreated control), with a higher abundance, also in shelf-life fruit (6.0% for the EO treatment, 1.8% for the chemical treatment and 13.2% for the untreated control). *Aspergillus* had a presence at harvest (5.2%). Also, *Fusarium* was detected during storage, with greater abundance in the untreated fruit (9.7%) compared to the chemical treatment (7.3%) and EO treatment (7.0%). In shelf life, the presence of this genus was lower in all considered treatments, but this time presented a lower abundance in untreated fruit (1.3%) compared to the chemical treatment (2.0%) and the EO treatment (1.5%).

## 4. Discussion

The antifungal activity of five EOs applied through biofumigation was assessed *in vitro* and *in vivo* on stored nectarines. The chemical compositions of the five essential oils were determined through the GC-MS technique in previous work [33]. Briefly, carvacrol was the major component of oregano EO (68.00%) but was also present in thyme EO (4.43%). On the other hand, thymol was the major component in thyme EO (43.30%), and a small amount was present in oregano EO (1.98%). For these two EOs, a large amount of γ-terpinene (7.73% and 9.91% for oregano and thyme EOs, respectively) was present. In basil, there was a high percentage of linalool (58.30%), while for lemon and fennel, the major components were limonene (66.90%) and trans-anethole (50.50%), respectively. Finally, p-cymene was present in all five EOs (8.04% for oregano EO, 18.95% for thyme EO, 0.23% for basil EO, 1.44% for lemon EO, and 1.53% for fennel EO). It is important to note the high number of components of the EOs: 28, 27, 41, 18, and 21 molecules for oregano, thyme, basil, lemon, and fennel EOs, respectively. The *in vitro* trials were performed at 25 °C as a preliminary test to verify the inhibitory potential of the essential oils at the optimal temperature for *Monilinia* spp. growth. The Petri dishes were sealed with Parafilm; therefore, therefore only small gas could be released, and the terpenes were more concentrated and active in inhibiting the pathogen growth. *In vitro* experiments showed a 100% inhibition of mycelial growth of *M. fructicola* for fennel (1.0% and 0.5%) and basil (1.0%) EOs, whose major constituents are t-anethole and linalool, respectively. Linalool was reported to have weak antifungal [51] and antioxidant activity [52]. On the other hand, Balsells-Llauradó et al. [53] demonstrated the antifungal activity *in vitro* of linalool against *M. laxa* depending on the concentration tested. Elshafie et al. [51] showed an important antifungal action for basil EO. This is because, as can be reasonably assumed considering the high number of molecules present, the action of EOs is due to a synergistic action between several, two, or more components and not to a single compound. In the basil EO used for this work, we found 41 different active compounds through GC-MS analysis. No data were found in the literature for the antifungal activity of t-anethole against *M. fructicola*, but this molecule showed good antifungal activity against *M. laxa* [54] and relevant antioxidant activity for the presence of an unsaturation conjugated with the aromatic ring [55].

Moreover, *in vitro* assays showed complete inhibition of the mycelial growth by thyme and oregano EOs, characterized by a phenolic monoterpenoid compound (thymol and carvacrol, respectively) as a major constituent. Thymol and carvacrol have important activity against fungal plant pathogens, attributed to their antioxidant activity [52], and stimulate the plant defenses [15,30,51]. Thymol vapors were reported to inhibit the mycelial growth of *M. fructicola* and to reduce conidia viability. In fact, thymol crystallizes on the external surface of the fungal cell walls, and the exposed structures are characterized by disrupted cell membranes and disorganized cytoplasmic organelles [35,56,57]. Furthermore, in both EOs, there was a non-negligible amount of γ-terpinene and p-cymene, non-phenolic monoterpenes. It has been shown that p-cymene may be able to facilitate the transport of thymol and carvacrol into the fungal cell by modifying the membranes, through membrane expansion, and by affecting the membrane potential of intact cells [58,59,60]. Instead, γ-terpinene can prolong the antifungal and antioxidant activity of the two phenolic compounds, starting with a process of autoxidation that generates the p-cymene. Subsequent hydroxylation in the ortho or meta position of p-cymene leads to the formation of carvacrol and thymol, respectively [28,61,62].

The five essential oils were further tested *in vivo* to select the most effective ones. Essential oils were used at higher concentrations than *in vitro* (2.0%), and their efficacy in the control of brown rot of nectarines was assessed. The evaluation of the rot incidence after 14 days of storage at 1 ± 1 °C confirmed the results obtained *in vitro* for fennel and basil EOs. In contrast, oregano and thyme EOs showed a rot incidence (17% and 20%, respectively) not significantly different from the inoculated control (20%). Therefore, the phenolic compounds were less effective in controlling brown rot *in vivo* on nectarines. This is probably due to the interaction of these molecules with the food matrix constituents, which could decrease their activity [60,63]. The results obtained for basil EO are partially consistent with Santoro et al. [35], who showed that savory EO (main components: linalool 22.16%, carvacrol 13.29%, and thymol 10.67%) was effective in the control of *M. fructicola*. Furthermore, it was observed, through SPME-GC-MS sampling, that in the storage atmosphere, the phenolic components were extremely low, whereas linalool was the main component at the three sampling times [35]. Studies performed on terpenoid biosynthetic pathways showed that linalool- and farnesal-related pathways were upregulated only in resistant tissues of nectarines, which suggests the role of linalool, present in basil EO, in mediating resistance against brown rot [53]. Although no previous data were found about the efficacy of fennel EO *in vivo* against *Monilinia* spp., the EO was effective *in vivo* against several fruit postharvest pathogens, such as *Botrytis cinerea* on grape [64] and *B. cinerea* and *P. expansum* on apples [29].

Basil, fennel, and lemon EOs were selected for setting up larger efficacy trials without pathogen inoculation. After 28 days of storage at 1 ± 1 °C, the three EOs tested were as effective as the chemical control in the control of nectarine rots. In both the screening and efficacy trials, at the end of shelf life, fruits were no longer exposed to EO vapors, and the rot incidence increased for all the fruits treated with EOs, and it was not statistically different from the untreated control. This confirmed the fungistatic and non-fungicidal activity of EOs, as previously reported [16,33]. However, the treatment with basil EO showed a lower rot incidence after shelf life (Figure 2), and it was selected to perform the microbiome analysis.

The presence of significant differences in the alpha diversity between epiphytic and endophytic communities, with a higher Shannon index and number of observed features for the epiphytes, as well as the stark difference in beta diversity, was previously observed and discussed in other studies performed with essential oils [16].

The microbiome composition showed that the treatment with basil EO was able to reduce the abundance of *Monilinia* spp. at the epiphytic level. The major component of basil EO, linalool, was found to have efficacy *in vitro* against *M. laxa*, and brown rot-resistant nectarine tissues previously showed to upregulate the associated biosynthetic pathway [53]. By contrast, *Penicillium* spp. was not negatively affected by basil EO. On the contrary, its abundance increased. *Penicillium expansum*, the causal agent of blue mold on nectarines, was previously shown to be weakly sensitive to basil EO treatment *in vivo* when inoculated on apples [33]. Previous microbiome analyses of fruits treated with EOs showed that *Penicillium* spp. is tolerant to other EO treatments [16]. It is, therefore, reasonable to assume that *Penicillium* spp. increased by occupying the ecological niche left by other fungal genera. Other fungal pathogens, such as *Diplodia*, *Podosphaera*, and *Botryosphaeria*, did not seem to be affected by the basil EO treatment. For *Diplodia*, this may be due to the presence of innate detoxification mechanisms, as at least one species showed to be able to degrade linalool to linalool oxide [65]. Concerning *Botryosphaeria*, linalool was previously shown to be less effective against this pathogen compared to other molecules such as thymol and carvacrol [66]. Regarding the higher abundance of *Podosphaera*, it must be highlighted that while some EOs were shown to inhibit the growth of this pathogen [67,68], none had linalool as the main component.

After storage, *Aureobasidium* spp. was present in larger amounts in epiphytic communities of basil-EO-treated fruits compared to both untreated control and chemical control. Since *Aureobasdium* spp. has been shown to catalyze the formation of linalool in sugar-rich substrates [69], it is reasonable to assume that higher levels of the molecule do not interfere with its development. Moreover, the use of *Aureobasidium* spp. as a biocontrol agent may be promising, as it has been reported to be effective against *Monilinia* spp. on peaches and nectarines, thanks to a combination of lytic enzyme secretion, production of bioactive volatile compounds, and competition for nutrient and space [5,70,71,72,73]. The higher presence of *Aureobasidium* spp. on nectarines treated with basil EO could explain the lower percentage of rot compared to other treatments and the untreated control. This suggests a possible synergistic effect of the yeast with the action of the EO and the compatibility of the treatments in the framework of an integrated disease control strategy.

Fruit quality parameters such as firmness, total soluble solids, and titratable acidity were analyzed at harvest, after storage, and at the end of shelf life. The results showed that EO vapors did not affect the overall quality, but treated fruits showed slightly higher firmness at the end of shelf life. Additionally, it has been reported that EO can reduce weight loss and preserve the content of ascorbic acid and carotenoids, which are generally subject to decrease during fruit ripening, as they are photosensitive and heat sensitive and easily oxidized if left unprotected from light and atmosphere [35,74].

No phytotoxic effects were observed in any treatment with EOs, neither during cold storage nor during shelf life, demonstrating that biofumigation can be a suitable method to apply these treatments. The release of EOs by biofumigation, in addition to preventing phytotoxic effects, does not significantly modify the organoleptic profile of the fruit, which could occur when the EOs are applied by direct contact [30,36].

## 5. Conclusions

Treatments with fennel, lemon, and basil EOs proved to be effective in reducing the postharvest diseases of nectarines during storage, preserving the quality of final products. Recent studies on basil EO have demonstrated the upregulation of linalool- and farnesal-related pathways in resistant nectarine tissues. This suggests that linalool, a component of basil EO, may contribute to resistance against brown rot. The application of EOs through biofumigation has been shown to be effective without causing side effects, such as phytotoxicity or alterations in organoleptic properties, showing potential for developing sustainable strategies for postharvest disease management. Further research is ongoing to optimize application methods and dosage. Moreover, EO could be used in synergy with other treatments as compatible biocontrol agents. Combining EO with other strategies could improve the overall effectiveness of disease management and extend the shelf life of the fruits. It should be emphasized that the efficacy of EOs is highly dependent on their chemical composition as well as several factors such as the crop, the target pathogens, the concentration of EOs used, and the environmental conditions. Establishing clear and standardized guidelines for EO application and regulation can ensure their safe and effective use. Additionally, fostering collaboration among researchers, industry stakeholders, and regulatory bodies can help address any challenges and promote the responsible utilization of EOs as a promising tool to control postharvest diseases of stone fruit.

## Figures and Tables

**Table 1:** Effect of EOs on mycelial growth (diameter, cm) of the strain 1539 of *M. fructicola* in sandwich plate experiments. EOs were applied at different concentrations (0.1, 0.5, and 1.0%). Petri dishes were maintained at 25 ± 1 °C for 28 days. Values at the same time point, followed by the same letter, are not significantly different by Duncan’s multiple range test (*p* < 0.05).

Treatment	Average Diameter (cm) ± SD	
	*14 days*	*28 days*
Thyme EO 1.0%	0.00 ± 0.00 ^a^	0.00 ± 0.00 ^a^
Thyme EO 0.5%	0.00 ± 0.00 ^a^	0.00 ± 0.00 ^a^
Thyme EO 0.1%	0.00 ± 0.00 ^a^	0.00 ± 0.00 ^a^
Basil EO 1.0%	0.00 ± 0.00 ^a^	0.00 ± 0.00 ^a^
Basil EO 0.5%	0.10 ± 0.02 ^a^	1.89 ± 0.05 ^b^
Basil EO 0.1%	1.25 ± 0.24 ^b^	3.52 ± 0.17 ^c^
Oregano EO 1.0%	0.00 ± 0.00 ^a^	0.00 ± 0.00 ^a^
Oregano EO 0.5%	0.00 ± 0.00 ^a^	0.00 ± 0.00 ^a^
Oregano EO 0.1%	0.00 ± 0.00 ^a^	0.66 ± 0.01 ^ab^
Fennel EO 1.0%	0.00 ± 0.00 ^a^	0.00 ± 0.00 ^a^
Fennel EO 0.5%	0.00 ± 0.00 ^a^	0.88 ± 0.06 ^ab^
Fennel EO 0.1%	2.06 ± 0.08 ^c^	2.93 ± 0.24 ^d^
Lemon EO 1.0%	1.89 ± 0.01 ^c^	3.98 ± 0.01 ^cd^
Lemon EO 0.5%	2.41 ± 0.01 ^d^	4.97 ± 0.27 ^d^
Lemon EO 0.1%	2.61 ± 0.35 ^de^	4.77 ± 0.17 ^d^
Control	2.86 ± 0.20 ^e^	4.18 ± 0.01 ^cd^

**Table 2:** Firmness, total soluble solids (TSSs), and titratable acidity (TA) of nectarines treated by biofumigation with EOs. Values are expressed as the mean of n = 3 replicates of 5 fruits ± standard deviation (SD). Values at the same time point, followed by the same letter, are not statistically different by Duncan’s multiple range test (*p* < 0.05). * Values are expressed as the mean of 3 replicates of 5 fruits ± standard deviation (SD).

Time Point (Temperature)	Treatment (Concentration)	Firmness [N/cm^2^] ± SD *	TSSs [%] ± SD *	TA [%] ± SD *
At harvest	-	82.07 ± 9.55	10.13 ± 1.27	0.49 ± 0.03
28 days of storage (1 ± 1 °C)	Untreated control	71.31 ± 11.78 ^a^	11.03 ± 1.02 ^a^	0.43 ± 0.10 ^a^
	Chemical control	75.49 ± 9.91 ^a^	11.80 ± 1.10 ^a^	0.43 ± 0.10 ^a^
	Basil EO (2.0%)	78.41 ± 10.91 ^a^	11.70 ± 1.65 ^a^	0.40 ± 0.06 ^a^
	Fennel EO (2.0%)	77.62 ± 10.02 ^a^	10.83 ± 1.50 ^a^	0.42 ± 0.05 ^a^
	Lemon EO (2.0%)	80.30 ± 9.37 ^a^	11.00 ± 0.17 ^a^	0.46 ± 0.08 ^a^
5 days of shelf life (20 ± 1 °C)	Untreated control	12.12 ± 3.36 ^a^	14.90 ± 0.72 ^a^	0.34 ± 0.09 ^a^
	Chemical control	16.34 ± 3.42 ^b^	13.43 ± 1.70 ^a^	0.30 ± 0.05 ^a^
	Basil EO (2.0%)	16.28 ± 3.95 ^b^	12.67 ± 0.55 ^a^	0.28 ± 0.02 ^a^
	Fennel EO (2.0%)	16.36 ± 4.10 ^b^	12.33 ± 2.08 ^a^	0.26 ± 0.03 ^a^
	Lemon EO (2.0%)	16.77 ± 2.35 ^b^	12.90 ± 0.62 ^a^	0.33 ± 0.00 ^a^

## Data Availability

Data are contained within the article and Supplementary Materials.

## Supplementary Materials

The following supporting information can be downloaded at https://www.mdpi.com/article/10.3390/jof10050341/s1. Table S1. Results of the Adonis analysis on the beta diversity distance matrix; parameter indicates the parameter or combination of parameters tested; F.model is the test statistic; R2 is the fraction of variance explained by the parameter; and Pr(>F) is the q-value, or FDR (false discovery rate) adjusted *p*-value. The rejection threshold was set at 0.05. NaN: not available. Table S2. Significance groups for the results of the pairwise PERMANOVA (permutational analysis of variance) and PERMDISP (permutational dispersion analysis) on the beta diversity distance matrix for all tissue/treatment/sampling time point combinations. The rejection threshold for the presence of significant differences was set at 0.05. Table S3. Results of the pairwise PERMANOVA (permutational analysis of variance) and PERMDISP (permutational dispersion analysis) on the beta diversity distance matrix for all tissue/treatment/sampling time point combinations. Pseudo-F and F-value are the PERMANOVA’s and PERMDISP’s statistics, respectively. Q-value is the FDR (false discovery rate) adjusted *p*-value.
