# Supplementary material for: Efficacy of Essential Oil Vapours in Reducing Postharvest Rots and Effect on the Fruit Mycobiome of Nectarines
Source: J Fungi (Basel). 2024 May 8;10(5):341. doi: 10.3390/jof10050341 (PMC11121902; doi:10.3390/jof10050341)
Supplement: Supplementary file 1 [file jof-10-00341-s001.zip › jof-2993320-supplementary.pdf]

Supplementary Materials

Efficacy of Essential Oil Vapours in Reducing Postharvest Rots and Effect on the Fruit Mycobiome of Nectarines

Giulia Remolif [1,2], Fabio Buonsenso [1,2], Giada Schiavon [1,2], Marco Garello [1,2], Davide Spadaro [1,2,*]

[1] Department of Agricultural, Forest and Food Sciences (DISAFA), University of Turin, Largo Paolo Braccini 2, 10095 Grugliasco (Italy)

[2] Interdepartmental Centre for the Innovation in the Agro-environmental Sector - AGROINNOVA, University of Turin, Largo Paolo Braccini 2, 10095 Grugliasco (Italy)

*Corresponding authors; email address: davide.spadaro@unito.it

Content.

**Table S1.** Results of the Adonis analysis on the beta diversity distance matrix. Parameter indicates the parameter or combination of parameters tested, F.model is the test's statistic, R2 is the fraction of variance explained by the parameter and Pr(>F) is the q-value, or FDR (false discovery rate) adjusted p-value. Rejection threshold was set at 0.05. NaN: not available.	**2**
**Table S2.** Significance groups for the results of the pairwise PERMANOVA (Permutational Analysis of Variance) and PERMDISP (Permutational Dispersion Analysis) on the beta diversity distance matrix for all tissue/treatment/sampling timepoint combinations. Rejection threshold for the presence of significant differences was set at 0.05.	**3**
**Table S3.** Results of the pairwise PERMANOVA (Permutational Analysis of Variance) and PERMDISP (Permutational Dispersion Analysis) on the beta diversity distance matrix for all tissue/treatment/sampling timepoint combinations. Pseudo-F and F-value are the PERMANOVA's and PERMDISP's statistics, respectively. Q-value is the FDR (false discovery rate) adjusted p-value.	**4**

Table S1. Results of the Adonis analysis on the beta diversity distance matrix. Parameter indicates the parameter or combination of parameters tested, F.model is the test's statistic, R2 is the fraction of variance explained by the parameter and Pr(>F) is the q-value, or FDR (false discovery rate) adjusted p-value. Rejection threshold was set at 0.05. NaN: not available.

**Parameter**	**F.model**	**R2**	**Pr(>F)**
Tissue	86.62	0.44	0.001
Treatment	10.34	0.11	0.001
Sampling timepoint	3.48	0.04	0.007
Tissue x Treatment	9.27	0.09	0.001
Tissue x Sampling timepoint	4.30	0.04	0.003
Treatment x Sampling timepoint	3.53	0.04	0.01
Tissue x Treatment x Sampling timepoint	0.06	<0.01	0.993
Residuals	0.65	0.24	NaN
Total	NaN	1	NaN

Table S2. Significance groups for the results of the pairwise PERMANOVA (Permutational Analysis of Variance) and PERMDISP (Permutational Dispersion Analysis) on the beta diversity distance matrix for all tissue/treatment/sampling timepoint combinations. Rejection threshold for the presence of significant differences was set at 0.05.

Group	PERMANOVA significance group	PERMADISP significance group
Endophytes, Harvest, Untreated control	a	a
Endophytes, Storage, Untreated control	ab	a
Endophytes, Storage, Chemical control	a	a
Endophytes, Storage, Basil EO	a	a
Endophytes, Shelf-life, Untreated control	a	a
Endophytes, Shelf-life, Chemical control	ab	a
Endophytes, Shelf-life, Basil EO	a	a
Epiphytes, Harvest, Untreated control	cde	a
Epiphytes, Storage, Untreated control	bcde	a
Epiphytes, Storage, Chemical control	g	a
Epiphytes, Storage, Basil EO	e	a
Epiphytes, Shelf-life, Untreated control	d	a
Epiphytes, Shelf-life, Chemical control	f	a
Epiphytes, Shelf-life, Basil EO	c	a

Table S3. Results of the pairwise PERMANOVA (Permutational Analysis of Variance) and PERMDISP (Permutational Dispersion Analysis) on the beta diversity distance matrix for all tissue/treatment/sampling timepoint combinations. Pseudo-F and F-value are the PERMANOVA's and PERMDISP's statistics, respectively. Q-value is the FDR (false discovery rate) adjusted p-value.

Group 1	Group 2	Sample size	PERMANOVA			PERMADISP		
			pseudo-F	p-value	q-value	F-value	p-value	q-value
Endophytes, Harvest, Untreated control	Endophytes, Shelf-life, Basil EO	9	0.29	0.890	0.920	0.93	0.431	0.643
Endophytes, Harvest, Untreated control	Endophytes, Shelf-life, Chemical control	8	0.10	0.889	0.920	0.04	0.847	0.914
Endophytes, Harvest, Untreated control	Endophytes, Shelf-life, Untreated control	10	0.06	0.918	0.928	0.13	0.667	0.788
Endophytes, Harvest, Untreated control	Endophytes, Storage, Basil EO	8	0.26	0.904	0.924	0.61	0.563	0.712
Endophytes, Harvest, Untreated control	Endophytes, Storage, Chemical control	9	2.99	0.098	0.127	0.05	0.864	0.914
Endophytes, Harvest, Untreated control	Endophytes, Storage, Untreated control	8	4.00	0.061	0.083	0.03	0.912	0.943
Endophytes, Harvest, Untreated control	Epiphytes, Harvest, Untreated control	10	11.91	0.006	0.033	0.02	0.822	0.902
Endophytes, Harvest, Untreated control	Epiphytes, Shelf-life, Basil EO	10	25.89	0.007	0.033	1.92	0.016	0.104
Endophytes, Harvest, Untreated control	Epiphytes, Shelf-life, Chemical control	10	27.75	0.013	0.033	3.48	0.009	0.100
Endophytes, Harvest, Untreated control	Epiphytes, Shelf-life, Untreated control	10	22.37	0.010	0.033	1.38	0.036	0.193
Endophytes, Harvest, Untreated control	Epiphytes, Storage, Basil EO	10	13.82	0.007	0.033	2.09	0.103	0.329
Endophytes, Harvest, Untreated control	Epiphytes, Storage, Chemical control	10	19.64	0.010	0.033	2.42	0.011	0.100
Endophytes, Harvest, Untreated control	Epiphytes, Storage, Untreated control	10	7.37	0.011	0.033	0.52	0.493	0.712
Endophytes, Shelf-life, Basil EO	Endophytes, Shelf-life, Chemical control	7	1.00	0.455	0.538	1.39	0.262	0.495
Endophytes, Shelf-life, Basil EO	Endophytes, Shelf-life, Untreated control	9	0.21	0.882	0.920	0.31	0.720	0.840
Endophytes, Shelf-life, Basil EO	Endophytes, Storage, Basil EO	7	0.13	0.945	0.945	0.00	0.973	0.991
Endophytes, Shelf-life, Basil EO	Endophytes, Storage, Chemical control	8	2.63	0.170	0.212	1.06	0.326	0.549
Endophytes, Shelf-life, Basil EO	Endophytes, Storage, Untreated control	7	4.15	0.059	0.081	2.06	0.348	0.566
Endophytes, Shelf-life, Basil EO	Epiphytes, Harvest, Untreated control	9	12.65	0.018	0.033	1.13	0.084	0.294
Endophytes, Shelf-life, Basil EO	Epiphytes, Shelf-life, Basil EO	9	55.03	0.013	0.033	0.19	0.392	0.605
Endophytes, Shelf-life, Basil EO	Epiphytes, Shelf-life, Chemical control	9	97.06	0.009	0.033	1.90	0.005	0.100

Table S3. (continued)

Group 1	Group 2	Sample size	PERMANOVA			PERMADISP		
			pseudo-F	p-value	q-value	F-value	p-value	q-value
Endophytes, Shelf-life, Basil EO	Epiphytes, Shelf-life, Untreated control	9	47.78	0.008	0.033	0.03	0.774	0.870
Endophytes, Shelf-life, Basil EO	Epiphytes, Storage, Basil EO	9	30.13	0.014	0.033	0.30	0.336	0.556
Endophytes, Shelf-life, Basil EO	Epiphytes, Storage, Chemical control	9	56.69	0.005	0.033	0.53	0.199	0.455
Endophytes, Shelf-life, Basil EO	Epiphytes, Storage, Untreated control	9	6.70	0.007	0.033	1.50	0.283	0.495
Endophytes, Shelf-life, Chemical control	Endophytes, Shelf-life, Untreated control	8	0.24	0.794	0.860	0.03	0.823	0.902
Endophytes, Shelf-life, Chemical control	Endophytes, Storage, Basil EO	6	0.88	0.607	0.674	0.83	0.251	0.486
Endophytes, Shelf-life, Chemical control	Endophytes, Storage, Chemical control	7	4.04	0.093	0.124	0.00	1.000	1.000
Endophytes, Shelf-life, Chemical control	Endophytes, Storage, Untreated control	6	4.91	0.116	0.149	0.28	0.524	0.712
Endophytes, Shelf-life, Chemical control	Epiphytes, Harvest, Untreated control	8	10.49	0.016	0.033	0.12	0.528	0.712
Endophytes, Shelf-life, Chemical control	Epiphytes, Shelf-life, Basil EO	8	35.30	0.017	0.033	4.48	0.180	0.455
Endophytes, Shelf-life, Chemical control	Epiphytes, Shelf-life, Chemical control	8	35.97	0.022	0.036	17.23	0.020	0.121
Endophytes, Shelf-life, Chemical control	Epiphytes, Shelf-life, Untreated control	8	26.86	0.018	0.033	1.85	0.211	0.455
Endophytes, Shelf-life, Chemical control	Epiphytes, Storage, Basil EO	8	18.81	0.017	0.033	4.54	0.219	0.455
Endophytes, Shelf-life, Chemical control	Epiphytes, Storage, Chemical control	8	23.88	0.014	0.033	4.64	0.105	0.329
Endophytes, Shelf-life, Chemical control	Epiphytes, Storage, Untreated control	8	5.23	0.058	0.081	0.51	0.560	0.712
Endophytes, Shelf-life, Untreated control	Endophytes, Storage, Basil EO	8	0.32	0.858	0.919	0.19	0.732	0.843
Endophytes, Shelf-life, Untreated control	Endophytes, Storage, Chemical control	9	2.88	0.127	0.161	0.03	0.879	0.919
Endophytes, Shelf-life, Untreated control	Endophytes, Storage, Untreated control	8	4.22	0.058	0.081	0.27	0.576	0.718
Endophytes, Shelf-life, Untreated control	Epiphytes, Harvest, Untreated control	10	11.78	0.010	0.033	0.24	0.392	0.605
Endophytes, Shelf-life, Untreated control	Epiphytes, Shelf-life, Basil EO	10	29.01	0.009	0.033	0.82	0.011	0.100
Endophytes, Shelf-life, Untreated control	Epiphytes, Shelf-life, Chemical control	10	34.79	0.004	0.033	1.88	0.008	0.100

Table S3. (continued)

Group 1	Group 2	Sample size	PERMANOVA			PERMADISP		
			pseudo-F	p-value	q-value	F-value	p-value	q-value
Endophytes, Shelf-life, Untreated control	Epiphytes, Shelf-life, Untreated control	10	25.10	0.009	0.033	0.52	0.058	0.245
Endophytes, Shelf-life, Untreated control	Epiphytes, Storage, Basil EO	10	14.88	0.008	0.033	0.95	0.084	0.294
Endophytes, Shelf-life, Untreated control	Epiphytes, Storage, Chemical control	10	23.30	0.008	0.033	1.19	0.014	0.104
Endophytes, Shelf-life, Untreated control	Epiphytes, Storage, Untreated control	10	6.99	0.010	0.033	0.91	0.384	0.605
Endophytes, Storage, Basil EO	Endophytes, Storage, Chemical control	7	2.18	0.191	0.235	0.65	0.225	0.455
Endophytes, Storage, Basil EO	Endophytes, Storage, Untreated control	6	2.99	0.098	0.127	1.31	0.274	0.495
Endophytes, Storage, Basil EO	Epiphytes, Harvest, Untreated control	8	10.47	0.016	0.033	0.75	0.233	0.461
Endophytes, Storage, Basil EO	Epiphytes, Shelf-life, Basil EO	8	48.11	0.016	0.033	0.20	0.467	0.685
Endophytes, Storage, Basil EO	Epiphytes, Shelf-life, Chemical control	8	86.96	0.024	0.038	1.56	0.201	0.455
Endophytes, Storage, Basil EO	Epiphytes, Shelf-life, Untreated control	8	42.18	0.021	0.036	0.04	0.654	0.785
Endophytes, Storage, Basil EO	Epiphytes, Storage, Basil EO	8	27.81	0.018	0.033	0.29	0.414	0.628
Endophytes, Storage, Basil EO	Epiphytes, Storage, Chemical control	8	51.13	0.020	0.036	0.47	0.219	0.455
Endophytes, Storage, Basil EO	Epiphytes, Storage, Untreated control	8	5..57	0..025	0.039	1.04	0.274	0.495
Endophytes, Storage, Chemical control	Endophytes, Storage, Untreated control	7	0.49	0.629	0.690	0.23	0.557	0.712
Endophytes, Storage, Chemical control	Epiphytes, Harvest, Untreated control	9	5.85	0.016	0.033	0.13	0.548	0.712
Endophytes, Storage, Chemical control	Epiphytes, Shelf-life, Basil EO	9	18.71	0.004	0.033	2.75	0.050	0.239
Endophytes, Storage, Chemical control	Epiphytes, Shelf-life, Chemical control	9	57.39	0.007	0.033	6.58	0.010	0.100
Endophytes, Storage, Chemical control	Epiphytes, Shelf-life, Untreated control	9	24.28	0.010	0.033	1.54	0.062	0.245
Endophytes, Storage, Chemical control	Epiphytes, Storage, Basil EO	9	11.52	0.008	0.033	2.97	0.056	0.245
Endophytes, Storage, Chemical control	Epiphytes, Storage, Chemical control	9	35.06	0.012	0.033	3.35	0.025	0.142
Endophytes, Storage, Chemical control	Epiphytes, Storage, Untreated control	9	4.47	0.031	0.046	0.66	0.317	0.544

Table S3. (continued)

Group 1	Group 2	Sample size	PERMANOVA			PERMADISP		
			pseudo-F	p-value	q-value	F-value	p-value	q-value
Endophytes, Storage, Untreated control	Epiphytes, Harvest, Untreated control	8	5.39	0.023	0.037	0.00	0.980	0.991
Endophytes, Storage, Untreated control	Epiphytes, Shelf-life, Basil EO	8	16.83	0.020	0.036	4.62	0.188	0.455
Endophytes, Storage, Untreated control	Epiphytes, Shelf-life, Chemical control	8	55.55	0.022	0.036	9.55	0.014	0.104
Endophytes, Storage, Untreated control	Epiphytes, Shelf-life, Untreated control	8	24.19	0.013	0.033	2.75	0.195	0.455
Endophytes, Storage, Untreated control	Epiphytes, Storage, Basil EO	8	12.98	0.018	0.033	4.77	0.172	0.455
Endophytes, Storage, Untreated control	Epiphytes, Storage, Chemical control	8	35.80	0.024	0.038	5.02	0.155	0.455
Endophytes, Storage, Untreated control	Epiphytes, Storage, Untreated control	8	4.47	0.058	0.081	0.24	0.541	0.712
Epiphytes, Harvest, Untreated control	Epiphytes, Shelf-life, Basil EO	10	0.57	0.521	0.600	2.16	0.103	0.329
Epiphytes, Harvest, Untreated control	Epiphytes, Shelf-life, Chemical control	10	31.05	0.008	0.033	3.64	0.006	0.100
Epiphytes, Harvest, Untreated control	Epiphytes, Shelf-life, Untreated control	10	5.71	0.051	0.075	1.63	0.060	0.245
Epiphytes, Harvest, Untreated control	Epiphytes, Storage, Basil EO	10	1.72	0.220	0.267	2.33	0.082	0.294
Epiphytes, Harvest, Untreated control	Epiphytes, Storage, Chemical control	10	16.74	0.018	0.033	2.65	0.006	0.100
Epiphytes, Harvest, Untreated control	Epiphytes, Storage, Untreated control	10	0.69	0.404	0.484	0.37	0.283	0.495
Epiphytes, Shelf-life, Basil EO	Epiphytes, Shelf-life, Chemical control	10	146.32	0.010	0.033	1.61	0.008	0.100
Epiphytes, Shelf-life, Basil EO	Epiphytes, Shelf-life, Untreated control	10	13.65	0.012	0.033	0.06	0.769	0.870
Epiphytes, Shelf-life, Basil EO	Epiphytes, Storage, Basil EO	10	8.62	0.022	0.036	0.03	0.858	0.914
Epiphytes, Shelf-life, Basil EO	Epiphytes, Storage, Chemical control	10	65.23	0.007	0.033	0.19	0.177	0.455
Epiphytes, Shelf-life, Basil EO	Epiphytes, Storage, Untreated control	10	0.50	0.532	0.605	2.35	0.045	0.228
Epiphytes, Shelf-life, Chemical control	Epiphytes, Shelf-life, Untreated control	10	48.39	0.012	0.033	1.23	0.015	0.104
Epiphytes, Shelf-life, Chemical control	Epiphytes, Storage, Basil EO	10	94.92	0.008	0.033	0.90	0.004	0.100
Epiphytes, Shelf-life, Chemical control	Epiphytes, Storage, Chemical control	10	11.75	0.011	0.033	0.23	0.588	0.723

Table S3. (continued)

Group 1	Group 2	Sample size	PERMANOVA			PERMADISP		
			pseudo-F	p-value	q-value	F-value	p-value	q-value
Epiphytes, Shelf-life, Chemical control	Epiphytes, Storage, Untreated control	10	8.98	0.007	0.033	3.12	0.182	0.455
Epiphytes, Shelf-life, Untreated control	Epiphytes, Storage, Basil EO	10	9.18	0.010	0.033	0.13	0.539	0.712
Epiphytes, Shelf-life, Untreated control	Epiphytes, Storage, Chemical control	10	15.52	0.011	0.033	0.32	0.501	0.712
Epiphytes, Shelf-life, Untreated control	Epiphytes, Storage, Untreated control	10	0.55	0.478	0.558	2.06	0.225	0.455
Epiphytes, Storage, Basil EO	Epiphytes, Storage, Chemical control	10	34.96	0.007	0.033	0.07	0.656	0.785
Epiphytes, Storage, Basil EO	Epiphytes, Storage, Untreated control	10	0.51	0.554	0.622	2.46	0.175	0.455
Epiphytes, Storage, Chemical control	Epiphytes, Storage, Untreated control	10	4.17	0.027	0.041	2.67	0.130	0.394
